# Omission of Completion Axillary Lymph Node Dissection for Patients with Breast Cancer Treated by Upfront Mastectomy and Sentinel Node Isolated Tumor Cells or Micrometastases

**DOI:** 10.3390/cancers16152666

**Published:** 2024-07-26

**Authors:** Gilles Houvenaeghel, Mellie Heinemann, Jean-Marc Classe, Catherine Bouteille, Pierre Gimbergues, Anne-Sophie Azuar, Marc Martino, Agnès Tallet, Monique Cohen, Alexandre de Nonneville

**Affiliations:** 1 Aix-Marseille Univ, CNRS, INSERM, Institut Paoli-Calmettes, Department of Surgical Oncology, CRCM, 13009 Marseille, France; 2 Centre Léon Bérard, 28 rue Laennec, 69673 Lyon, France; mellie.heinemann@lyon.unicancer.fr; 3 Institut René Gauducheau, Site Hospitalier Nord, 44800 St Herblain, France; jean-marc.classe@ico.unicancer.fr; 4 Institut Paoli-Calmettes, Department of Surgical Oncology, CRCM, 13009 Marseille, France; laurentc2@ipc.unicancer.fr (C.B.); cohenm@ipc.unicancer.fr (M.C.); 5 Centre Jean Perrin, 58 rue Montalembert, 63003 Clermont Ferrand, France; pierre.gimbergues@cjp.fr; 6 Hôpital de Grasse, Chemin de Clavary, 06130 Grasse, France; as.azuar@ch-grasse.fr; 7 Hôpital Saint Joseph, 26 Bd de Louvain, 13008 Marseille, France; martinom@ipc.unicancer.fr; 8 Institut Paoli-Calmettes, Department of Radiotherapy, CRCM, 13009 Marseille, France; richarda@ipc.unicancer.fr; 9 Aix-Marseille Univ, CNRS, INSERM, Institut Paoli-Calmettes, Department of Medical Oncology, CRCM, 13009 Marseille, France; tassindenonnevillea@ipc.unicancer.fr

**Keywords:** early breast cancer, sentinel lymph node, micrometastases, axillary lymph node dissection

## Abstract

**Simple Summary:**

Omission of completion axillary lymph node dissection (cALND) in patients undergoing mastectomy with sentinel node (SN) isolated tumor cells (ITC) or micrometastases is discussed. We evaluated the impact of cALND omission on survival in breast cancer (BC) patients treated by mastectomy with SN ITC or micrometastases. Among 554 BC, the non-SN involvement rate was 13.2%. With a median follow-up of 66.46 months, multivariate analysis showed that cALND omission was significantly associated with overall survival (OS, HR: 2.583, *p* = 0.043), disease-free survival (DFS, HR: 2.538, *p* = 0.008), and metastasis-free survival (MFS, HR: 2.756, *p* = 0.014). For Her2-positive or triple-negative patients, DFS was significantly impacted by cALND omission (HR: 38.451, *p* = 0.030). In ER-positive Her2-negative patients, DFS, OS, RFS, and MFS were also significantly affected (HR: 2.358, 3.317, 2.538, 2.756). For 161 patients ≤ 50 years with ER-positive/Her2-negative BC, OS and BCSS were notably associated with cALND omission (HR: 103.47 and 50.874). These findings suggest a negative prognostic impact of cALND omission in patients with SN micrometastases or ITC.

**Abstract:**

Omission of completion axillary lymph node dissection (cALND) in patients undergoing mastectomy with sentinel node (SN) isolated tumor cells (ITC) or micrometastases is debated due to potential under-treatment, with non-sentinel node (NSN) involvement detected in 7% to 18% of patients. This study evaluated the survival impact of cALND omission in a cohort of breast cancer (BC) patients treated by mastectomy with SN ITC or micrometastases. Among 554 early BC patients (391 pN1mi, 163 ITC), the NSN involvement rate was 13.2% (49/371). With a median follow-up of 66.46 months, multivariate analysis revealed significant associations between cALND omission and overall survival (OS, HR: 2.583, *p* = 0.043), disease-free survival (DFS, HR: 2.538, *p* = 0.008), and metastasis-free survival (MFS, HR: 2.756, *p* = 0.014). For Her2-positive or triple-negative patients, DFS was significantly affected by cALND omission (HR: 38.451, *p* = 0.030). In ER-positive Her2-negative BC, DFS, OS, recurrence-free survival (RFS), and MFS were significantly associated with cALND omission (DFS HR: 2.358, *p* = 0.043; OS HR: 3.317; RFS HR: 2.538; MFS HR: 2.756). For 161 patients aged ≤50 years with ER-positive/Her2-negative cancer, OS and breast cancer-specific survival (BCSS) were notably impacted by cALND omission (OS HR: 103.47, *p* = 0.004; BCSS HR: 50.874, *p* = 0.035). These findings suggest a potential negative prognostic impact of cALND omission in patients with SN micrometastases or ITC. Further randomized trials are needed.

## 1. Introduction

Sentinel lymph node biopsy (SLNB) has been the standard for axillary staging in clinically node-negative breast cancer patients undergoing upfront surgery since the NSABP B-32 trial demonstrated its efficacy in cT1-2 N0 breast cancer (BC) [1]. Studies have shown that SLNB alone results in lower morbidity, particularly lower rates of lymphedema [2,3,4], compared to axillary lymph node dissection (ALND) or completion ALND (cALND) after SLNB. Additionally, patient-reported outcomes indicate reduced arm morbidity with SLNB alone, positively affecting quality of life [3,5,6,7,8].

Next, validation was achieved for cALND omission in BC with one or two involved SN by micro- or macrometastases without extensive capsular rupture and breast conservative treatment with adjuvant chemotherapy and/or endocrine therapy and radiotherapy [4], and for cALND omission in BC with involved SN by micrometastases with BCS or mastectomy [9]. In these two trials, the indication for SLNB was limited to tumors <50 mm. Axillary surgical de-escalation continues with recent preliminary results from two randomized trials [10,11] and pending results from other randomized trials [12,13,14].

For patients treated with upfront mastectomy and SN isolated tumor cells (ITC: pN0(i+) sn) or micrometastases (pN1mi sn), evidence for cALND omission remains limited due to underrepresentation in the IBCSG 23-01 trial [9]. In the SENOMIC trial, patients with SN micrometastases underwent BCS or mastectomy without cALND, yet the risk of involved non-sentinel nodes (NSN) remains significant, exposing patients to undertreatment. Adjuvant chemotherapy (AC) and postmastectomy radiotherapy (PMRT) with regional nodal irradiation (RNI) are often not indicated for these patients, unlike those with NSN involvement where these treatments are typically recommended.

This study aimed to evaluate the survival impact of cALND omission in a large cohort of BC patients treated with upfront mastectomy and SN ITC or micrometastases, considering tumor subtypes and age subgroups.

## 2. Material and Methods

From a large multicenter cohort, early BC patients who underwent upfront mastectomy in 13 French cancer centers between 1990 and 2023 were retrospectively reviewed and we selected those with pN0(i+) or pN1mi LN metastases.

The main prospectively recorded characteristics were: age (≤40 years, 41–50, 51–74, ≥75), tumor histology (ductal, lobular, mixt, other), SBR grade 1 or 2 or 3, sentinel node (SN) status (pN0(i+) or micrometastases), pT size (pT1, pT2 ≤ 30 mm, pT2 > 30 mm, pT3), lympho-vascular invasion (LVI), axillary surgery (sentinel lymph node biopsy (SLNB) or SLNB and completion axillary lymph node dissection (cALND)), NSN involvement at cALND, tumor subtypes, AC and PMRT.

Endocrine receptors (ER) were positive if either or both estrogen and progesterone receptors were positive, with a 10% positive tumor nuclei threshold and Her2 status was considered positive if positive by fluorescence in situ hybridization or immunohistochemistry scored at 3+.

Multivariate regression analyses were used to determine significant factors associated with cALND and radiotherapy.

Overall survival (OS) was determined by months elapsed between mastectomy and death of any cause, disease-free survival (DFS) by months elapsed between mastectomy and death of any cause or recurrence, relapse-free survival (RFS) by months elapsed between mastectomy and recurrence, metastasis-free survival (MFS) by months elapsed between mastectomy and metastases, breast-cancer specific survival (BCSS) by months elapsed between mastectomy and death associated with recurrence.

Survival analysis was performed for ER-positive Her2-negative BC patients ≤ 50 years and >50 years. Menopausal status was not recorded. For patients ≤ 50 years old, AC is usually administered when NSN at cALND is involved by macrometastases. For patients > 50 years, AC is administered according to clinical, histological, and genomic risk factors.

### Statistics

Standard descriptive statistics were used to describe patient and tumor characteristics. All statistical tests were two sided. The level of statistical significance was set at a *p*-value ≤ 0.05. Statistical analyses were performed using the SPSS 16.0 (SPSS Inc., Chicago, IL, USA).

## 3. Results

### 3.1. Population

Five hundred fifty-four patients met the inclusion criteria. The median age of the whole cohort was 54.0 years; (60.3% of patients (334/554) were >50 years. Characteristics of 554 early BC patients who underwent upfront mastectomy, with 391 pN1mi sentinel nodes and 163 pN0(i+) are shown in Table 1. Higher rates of lobular histology, lower rates of AC, lower rates of PMRT, and lower rates of endocrine therapy were found in pN0(i+)sn patients.

The characteristics of the 554 patients treated either by cALND or SLNB alone are shown in Table 2. Median ages were 60.0 years and 53.0 years for SLNB alone and SLNB with cALND, respectively.

Distribution between pN0(i+)sn and pN1mi sn was 66 pN0(i+)sn and 150 pN1mi sn for patients 50 years or lower, 97 pN0(i+)sn and 241 pN1mi sn for patients > 50 years (*p* = 0.640). For patients without cALND, the pN0(i+)sn rate was 48.5% (32/66) and 34.2% (40/117) for patients 50 years or lower and >50 years, respectively (*p* = 0.057). For patients with cALND, the pN0(i+)sn rate was 22.7% (34/150) and 25.8% (57/221) for patients > 50 years, respectively (*p* = 0.492). For patients with cALND, pN1 with macro metastases rates were 11.3% (17/150) and 14.5% (32/121), pN1 mi rates were 66.7% (100/150) and 62.4% (138/221) and pN0(i+) rates were 22.0% (33/150) and 23.1% (51/221), for patients 50 years or lower and >50 years, respectively (*p* = 0.618). 

pN1mi, grade 2 and 3, age 50.1 to 74.9, LVI, radiotherapy, AC were significantly associated with cALND (Table 3).

SLNB alone was significantly associated with less radiotherapy (OR: 0.503, *p* = 0.0006) and pT4 stage, LVI, pN1mi, AC were significantly associated with more radiotherapy (Table 4). In ER-positive, HER2-negative BC patients, PMRT, administered in 65% of patients (68% in those ≤50 years and 62.5% in those >50 years), was more often considered in an increasing burden of SLN metastases and in patients submitted to a cALND.

For BC patients Her2-positive and triple negative (TN), the PMRT rate was 72.7% (56/77): 55.6% (15/27) and 82.0% (41/50) for pN0(i+) and pN1mi (*p* = 0.013), respectively, 45.0% (9/20) and 82.5% (47/57) for SLNB alone and SLNB with cALND (*p* = 0.001), respectively.

### 3.2. Survival Analysis for All Patients

Median follow-up was 66.46 months for all patients, 41.011 and 75.00 for SLNB alone and SLNB with cALND, respectively. Results of OS, DFS, RFS and MFS in univariate analysis for all patients, and for SLNB alone and SLNB with cALND are reported in Table 5. In univariate analysis, pN1mi versus pN0(i+) (*p* = 0.027), no radiotherapy versus radiotherapy (*p* = 0.020) and pN status (*p* = 0.053) including cALND significantly impacted patients’ outcomes. Others criteria, tumor histology, grade, ER Her2 status, pT size, age, LVI, AC and endocrine therapy were non-significant.

**Figure 1:**
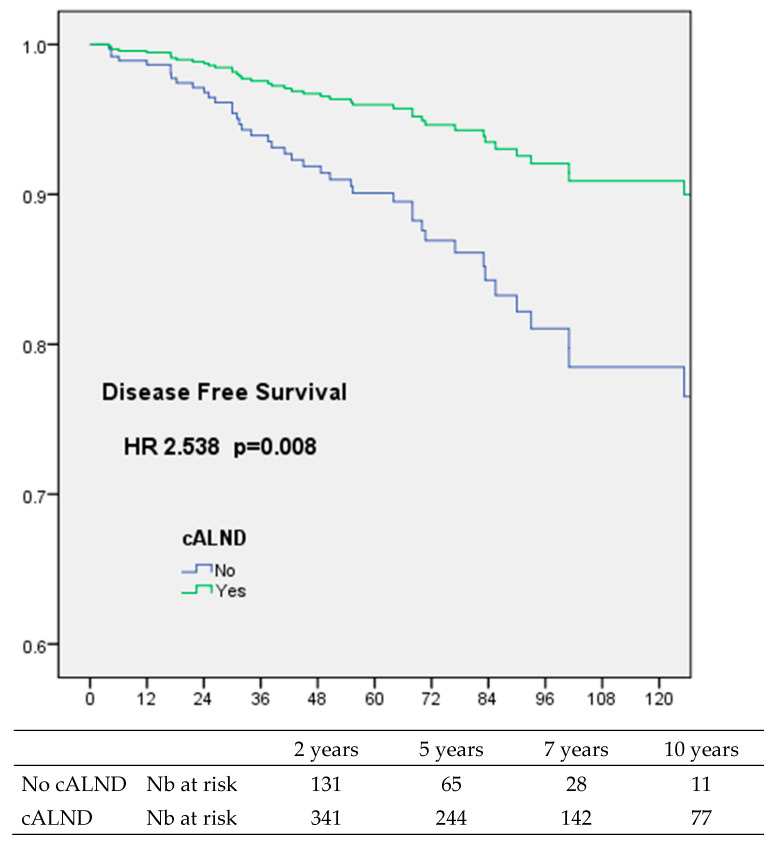
Disease-free survival for all patients according to completion axillary lymph node dissection (cALND) or not, in multivariate analysis.

Multivariate survival analyses were adjusted on significant factors in univariate survival analysis and significant factors associated with cALND and radiotherapy. In multivariate Cox analysis, only omission of cALND was significantly associated with OS (HR: 2.583, CI 95% 1.031–6.473, *p* = 0.043), DFS (HR: 2.538, CI 95% 1.276–5.049, *p* = 0.008) (Figure 1), RFS (HR: 2.565, CI 95% 1.204–5.463, *p* = 0.015) and MFS (HR: 2.756, CI 95% 1.228–6.183, *p* = 0.014) (Table 6). RFS was also negatively associated with no radiotherapy (HR: 2.342, CI 95% 1.047–5.239, *p* = 0.038) and NAC (17 patients, HR: 5.389, CI 95% 1.109–26.186, *p* = 0.037).

### 3.3. Survival Analysis According to pN1mi or pN0(i+) for All Patients

In univariate analysis, DFS was significantly higher for pN1mi in comparison with pN0(i+) (*p* = 0.027). In multivariate Cox analysis, OS, DFS, RFS and MFS was not significantly different between pN1mi versus pN0(i+) (Table 6).

### 3.4. Survival Analysis According to Tumor Subtypes

**Figure 2:**
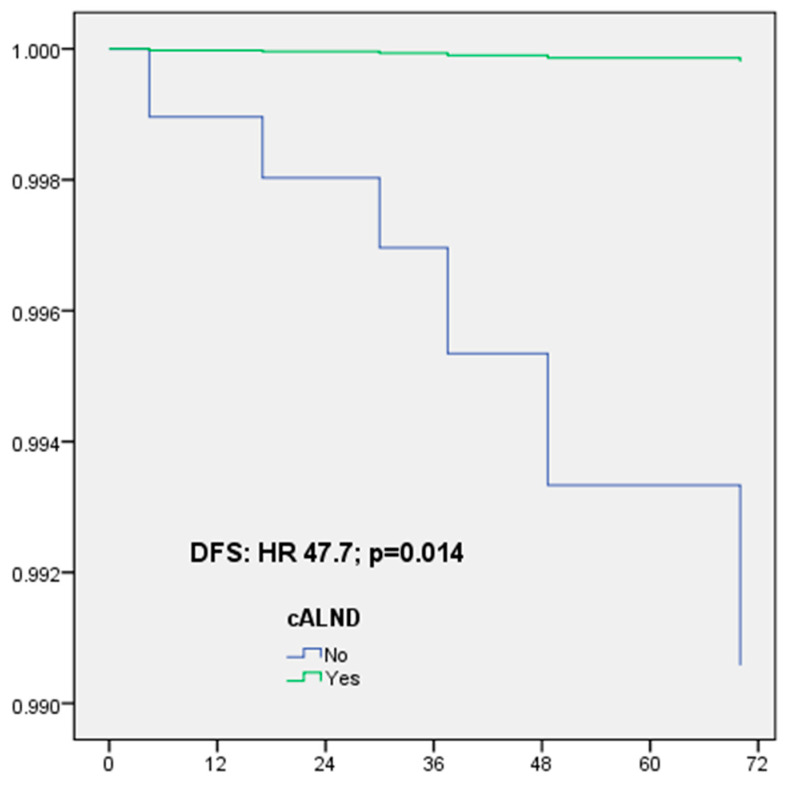
Disease-free survival (DFS) for Her2-positive or triple-negative breast cancer according to completion axillary lymph node dissection (cALND) or not, in multivariate analysis.

The number of patients with Her2-positive or TN BC was 77: cALND was performed for 57 patients (74.0%) and the NSN involvement rate with macrometastases was 17.5% (10/57). For 77 patients with Her2-positive or TN BC, DFS in multivariate analysis was significantly associated with omission of cALND (HR: 38.451, CI 95% 1.437–1028, *p* = 0.030) and no radiotherapy (HR: 7.824, CI 95% 1.246–49.118, *p* = 0.028) (Table 7, Figure 2).

**Figure 3:**
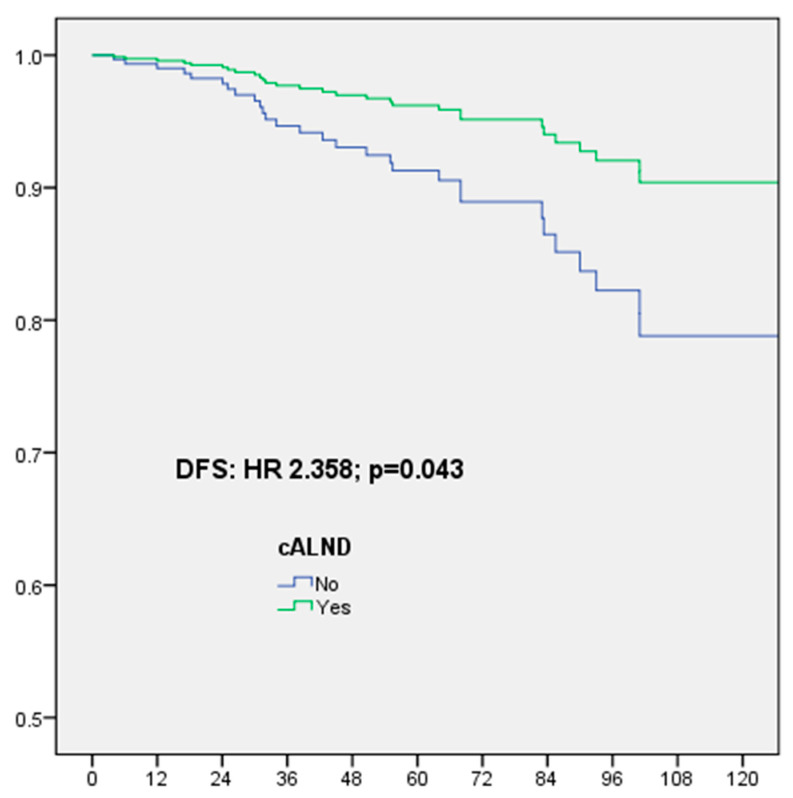
Disease-free survival (DFS) for ER-positive Her2-negative breast cancer according to completion axillary lymph node dissection (cALND) or not, in multivariate analysis.

The number of patients with ER-positive Her2-negative BC was 390: cALND was performed for 243 patients (62.3%) and the NSN involvement rate with macrometastases was 13.6% (33/243): 9.4% (10/106) and 16.8% (23/137) for patient’s ≤ 50 years and >50 years, respectively (*p* = 0.950). For 390 patients with ER-positive Her2-negative BC, in multivariate analysis, DFS was significantly associated only with omission of cALND (HR: 2.358, CI 95% 1.027–5.414, *p* = 0.043), OS was significantly associated with omission of cALND (HR: 3.317, CI 95% 1.054–10.439, *p* = 0.040) and AC (HR: 0.271, CI 95% 0.075–0.978, *p* = 0.046), RFS was significantly associated with omission of cALND (HR: 2.538, CI 95% 1.005–6.414, *p* = 0.049), NAC (HR: 8.232, CI 95% 1.223–55.409. *p* = 0.030) and no radiotherapy (HR: 2.342, CI 95% 1.047–5.239, *p* = 0.038), MFS was significantly associated with omission of cALND (HR: 2.756, CI 95% 1.228–6.183, *p* = 0.014) (Table 8, Figure 3).

**Figure 4:**
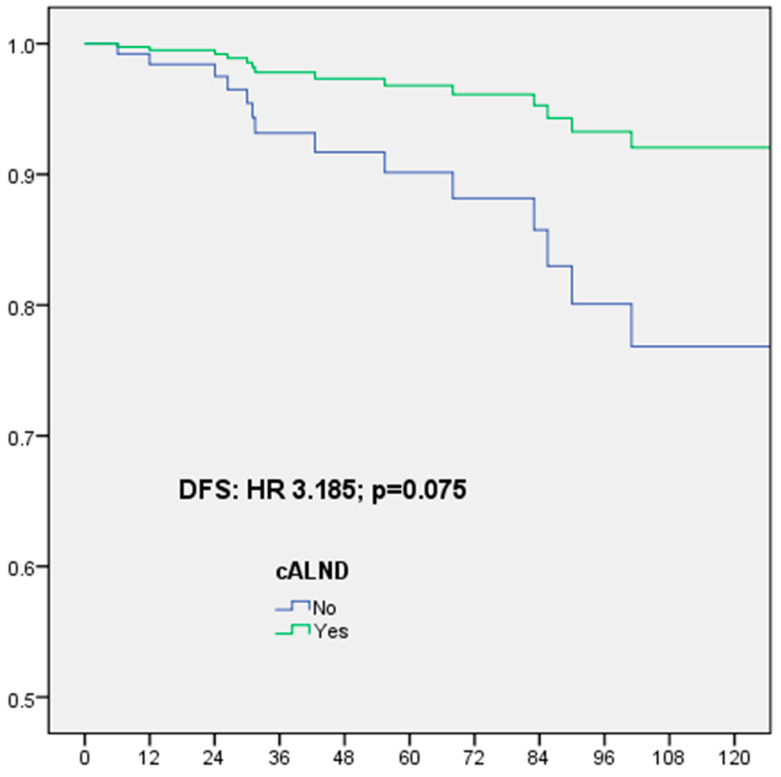
Disease-free survival (DFS) for ER-positive Her2-negative breast cancer patients ≤ 50 years according to completion axillary lymph node dissection (cALND) or not, in multivariate analysis.

In multivariate analysis, for 161 patients ≤ 50 years with ER-positive Her2-negative BC, DFS was significantly associated with no radiotherapy (51 patients) (HR: 3.948, CI 95% 1.016–15.335, *p* = 0.047) and result for omission of cALND was non-significant but with HR: 3.185, CI 95% 0.890–11.402, *p* = 0.075, OS was significantly associated with omission of cALND (HR: 103.47, CI 95% 4.583–2335.8, *p* = 0.004), grade 2 (HR: 0.055, CI 95% 0.005–0.614, *p* = 0.018), no radiotherapy (HR: 10.904, CI 95% 1.410–84.315, *p* = 0.022) and LVI (HR: 10.804, CI 95% 0.833–140.21, *p* = 0.022), BCSS was significantly associated with omission of cALND (HR: 50.874, CI 95% 1.330–1945.4, *p* = 0.035) (Table 9, Figure 4).

For 229 patients > 50 years with ER-positive Her2-negative BC, DFS in multivariate analysis was significantly associated with grade 3, LVI and neo-adjuvant chemotherapy (5 patients). Omission of cALND was non-significant: HR: 1.321, CI 95% 0.371–4.699, *p* = 0.667 for DFS, and OS, RFS, MFS and BCSS (Table 9).

## 4. Discussion

In this retrospective study, we report a non-sentinel involvement rate of 13.2% for patients treated by mastectomy with cALND after identification of SN micrometastases or isolated tumor cells. The PMRT rate was 64.9% (253/390): 48.3% (71/147) and 74.9% (182/243) for SLNB alone and SLNB with cALND (*p* < 0.0001), respectively. For all patients, in multivariate analysis, only omission of cALND was significantly associated with OS (HR: 2.583, *p* = 0.043) and DFS (HR: 2.538, *p* = 0.008). For Her2-positive or triple-negative BC patients, DFS in multivariate analysis was significantly associated with omission of cALND (HR: 38.451, *p* = 0.030). For ER-positive Her2-negative BC patients, in multivariate analysis, DFS was significantly associated only with omission of cALND (HR: 2.358, *p* = 0.043), OS, RFS, and MFS were significantly associated with omission of cALND (HR: 3.317, *p* = 0.040; HR: 2.538, *p* = 0.049; HR: 2.756, *p* = 0.014, respectively). OS and BCSS were significantly associated with omission of cALND for patients ≤ 50 years with ER-positive Her2-negative BC.

### 4.1. Survival Results and Axillary Recurrence Rates

Results of ACOSOG Z0011 trial demonstrate equivalent survival results between SLNB alone (436 patients) and SLNB with cALND (420 patients) for early BC with 1 or 2 SN micrometastases (301 patients) or macrometastases treated by BCS, adjuvant chemotherapy and or endocrine therapy, and whole breast radiotherapy [4]. However, a substantial axillary irradiation with high tangential irradiation fields, which can control the residual tumor burden (27.3% in cALND arm) was delivered in 18.9% of patients [15]. Only one nodal axillary recurrence was observed in a patient in the SLNB alone arm and none in the cALND arm.

Preliminary results of the SENOMAC trial [10] show no statistical RFS difference (median follow-up: 46.8 months) between cALND and ALND omission for early BC patients with macrometastases treated by BCS (n = 1620) or mastectomy (n = 920). The primary end-point was OS. The non-sentinel lymph node involvement rate was 34.5%. Radiotherapy was performed in 89.9% and 88.4% of patients in the SLNB alone arm and in the cALND arm, respectively.

The IBCSG 23-01 trial [9] included 934 patients with SN micrometastases or isolated tumor cells randomized between SLNB alone (453 patients) and SLNB with cALND (447 patients) for early BC treated by BCS or mastectomy with or without adjuvant chemotherapy and or endocrine therapy, and radiotherapy. Equivalent survival results were reported. However, few patients were treated by mastectomy (9.5%: n = 86) including 42 patients without radiotherapy, and cALND: 5.8% (5/86) received PMRT. After a 10-year follow-up, ipsilateral axillary recurrence rates were 1.7% (8/467) in SLNB alone arm and 0.4% (2/464) in cALND arm: 5 of 80 patients (6.3%) treated by BCS with intraoperative radiotherapy without cALND had an ipsilateral axillary recurrence. There were two axillary recurrences (2%) among the 96 patients treated by mastectomy.

The AATRM trial [16] included 233 patients with SN micrometastases randomized between SLNB alone (121 patients) and SLNB with cALND (112 patients): 225 treated by BCS and whole breast irradiation and 18 treated by mastectomy. At 5 years, the DFS rate was 98.2 percent for all patients without a statistically significant difference between the two groups. The axillary recurrence rate was 1.6% (2/121) in the SLNB alone arm: 1 patient treated by BCS without cALND (1/113: 0.9%) and 1 patient treated by mastectomy without cALND (1/8: 12.5%).

The SENOMIC trial [17] included patients with SN micrometastases treated by SLNB alone and BCS (349 patients) or mastectomy (217 patients: 38.3%). Patients who had mastectomy had significantly larger and higher-grade tumors than those operated with BCS and were more often in the youngest and oldest age groups. PMRT was performed in 30.9% of patients (67/217) and adjuvant chemotherapy in 55.8% (121/217). Patients who underwent mastectomy had a lower crude 3-year event-free survival rate than those treated by BCS (93.8 versus 97.8 percent, *p* = 0.011). On univariate analysis, patients who had mastectomy without adjuvant radiotherapy had a significantly higher risk of recurrence than those treated by BCS (HR: 2.91, CI 95% 1.25–6.75). Four isolated axillary recurrences were diagnosed in 217 patients after mastectomy (1.8%) of whom one had loco-regional irradiation and in 1 of 349 after BCS (0.3 per cent) (*p* = 0.054).

In summary, the axillary recurrence rate was 1% (16/1590: CI 95% 0.52–1.50) for cumulative results of IBCSG 23-01 trial (pN1mi and pN0(i+): 8/467) [9], AATRM trial (pN1mi: 2/121) [16], SENOMIC trial (pN1mi: 5/566) [17] and ACOSOG Z0011 trial (pN1mi: 1/436) [4]. The axillary recurrence rate for mastectomy was 2.2% (7/321: CI 95% 0.58–3.78) for cumulative results of these trials [4,9,16,17] and 3% (12/401: CI 95% 1.32–4.66) including 5 axillary recurrences among 80 patients treated by BCS with partial intraoperative radiotherapy in IBCSG 23-01 trial [9]. In a previous study [18], among 14,095 patients, the axillary recurrence rate was 0.51% and in multivariate analysis, the occurrence of axillary recurrence was significantly correlated with grade 2 or 3 BC, absence of radiotherapy and BC subtype (ER-negative Her2-positive). Axillary recurrence rates were 1% for triple-negative BC, 2.8% for HER2-positive BC, 0.4% for luminal A BC, 0.9% for HER2-negative luminal B BC, and 0.5% for HER2-positive luminal B BC. Survival in patients with axillary recurrence was significantly lower in the case of early-onset (2 years) axillary recurrence (*p* = 0.017).

### 4.2. The Non-Sentinel Nodes Involvement Rate

The involved non-sentinel nodes rate was 7% to 18%. These rates were 12.7% (59/464) in IBCSG 23-01 trial [9], 13.4% (15/112) in AATRM trial [16], 7.3% (12/164) in ACOSOG Z0011 trial for 164 patients with SN micrometastases [4], and 12.8% (152/1188) in a French cohort of patients with SN micrometastases [18]. In the study published by Tvedskov et al. [19], the rates of involved NSN were 16.5% (311/1881) and 7.4% (36/484) in two cohorts: 9.4% for SN ITC (28/299) and 17.9% (273/1521) for SN micrometastases. In a previous study, we reported positive NSN rates of 13.9% (40/287) for ITC and 14.1% (93/658) for pN1mi SN [20,21].

In the SERC trial [22], NSN involvement rates were 10.3% (22/214) for the first 1855 patients randomized with SN micrometastases treated by BCS (n = 388) or mastectomy (n = 82) (excluding pN0(i+)sn): 4.4% (4/92) without chemotherapy, 6.9% (2/29) with AC administered before cALND and 17.7% (15/85) with AC administered after cALND [13].

In summary, the NSN involvement rate was 13.77% (607/4407: CI 95% 12.76–14.79) for pN1mi and pN0(i+) [4,9,13,16,18,19,22]: 7.00% (86/1227: CI 95% 5.58–8.44) for ITC [13,19,20,22] and 15.28% (474/3101: CI 95% 14.02–16.55) for pN1mi [4,13,16,19,20,22]. These rates were in our study specifically dedicated to mastectomy 9.4% for all patients, 11.3% for pN1mi and 4.9% for pN0(i+). This pN stage under-evaluation may lead to under-treatment (less PMRT and RNI, and adjuvant chemotherapy particularly for pre-menopausal patients), with a possible negative impact on survival.

In a SEER database population [23], early BC patients with SN micrometastases treated by BCS, were compared according to the axillary surgery (SLNB alone and SLNB with cALND). Using a propensity score matched analysis, there was no difference in survival between patients who underwent axillary dissection and those who had SLNB alone.

No comparative survival results between SLNB alone and SLNB with cALND are available in the literature specifically for patients pN1mi treated by mastectomy without axillary radiotherapy. However, it was reported that, on Berg level 1, PMRT gives a dose at least equivalent to the one given by post-breast-conserving surgery radiotherapy [24].

### 4.3. Proportion of Tumor Subtypes

The proportion of ER-negative BC was low between 8.2% and 16.3% in literature studies [9,13,16,17,19,20,21,22,23] and 6.7% in our study. The proportion of Her2-positive BC was between 7.9% and 12.8% in literature studies [9,13,16,17,19,20,21,22,23] and 13.7% in our study. The proportion of TN BC was between 5.4% and 6.46% in literature studies [13,22,23] and 2.8% in our study. The proportion of ER-positive Her2-negative BC was between 83.07% and 86.7% in literature studies [13,22,23] and 83.5% in our study. Recurrence in ER-positive Her2-negative BC may develop after a long time, probably especially relevant in micrometastatic disease. For patients with micrometastases SN and BCS, different survival results between SLNB alone and cALND appear after 5-year follow-up [18].

Limitations: The main limitation is the retrospective design of this study. Despite multivariate analysis adjusted on numerous criteria, several biases can persist in the comparison between SLNB alone and SLNB with cALND. These results underline a possible negative prognostic effect of cALND omission in patients with SN micrometastases or isolated tumor cells. Consequently, results of randomized trials are required to demonstrate non-inferior results of cALND omission in comparison with cALND. In SENOMIC trial [17], there was no randomization, and all patients were treated without cALND by BCS or mastectomy. Previous randomized trials included very few patients with SN micrometastases treated by mastectomy [9,16].

In the non-inferiority POSNOC trial [12], with randomization between cALND or not, the main objective was 5-year axillary recurrence in patients with 1 or 2 macrometastases. The Dutch BOOG 2013-07 trial [25] will investigate whether completion axillary treatment can be safely omitted in SLN-positive breast cancer patient’s cT1-2 N0 treated with mastectomy with 1 to 3 SLN macrometastases.

In the non-inferiority SERC trial [13], with randomization between cALND or not, a stratification between SN micrometastases or isolated tumor cells and SN macrometastases was realized. The main objective was DFS. External validation of patients with SN micrometastases included in SERC trial was reported [22]. It is the only trial that can answer this situation. We hope to report the first survival results in the next months.

## 5. Conclusions

In this retrospective study, we report a non-sentinel involvement rate of 13.2% for patients treated by mastectomy with cALND after identification of SN micrometastases or isolated tumor cells. For all patients, in multivariate analysis, only the omission of cALND was significantly associated with OS (HR: 2.583) and DFS (HR: 2.538). For Her2-positive or triple-negative BC patients, DFS in multivariate analysis was significantly associated with omission of cALND (HR: 38.451, *p* = 0.030). For ER-positive Her2-negative BC patients, in multivariate analysis, DFS, OS, RFS and MFS were significantly associated with omission of cALND. OS and BCSS were significantly associated with omission of cALND for patients ≤ 50 years with ER-positive Her2-negative BC. These results underline a possible negative prognostic impact of cALND omission in patients with SN micrometastases or isolated tumor cells. Consequently, results of randomized trials are required to demonstrate non-inferior results of cALND omission in comparison with cALND.

## Figures and Tables

**Table 1:** Characteristics of 554 early breast cancer patients who underwent upfront mastectomy, with 391 pN1mi sentinel node and 163 pN0(i+).

		All Patients		pN1mi		pN0(i+)		Chi 2
		Nb	%	Nb	%	Nb	%	*p*
All patients		554		391	70.6	163	29.4	
Age	≤40	81	14.6	54	13.8	27	16.6	0.157
	41–50	139	25.1	98	25.1	41	25.2	
	51–74.9	263	47.5	181	46.3	82	31.2	
	≥75	71	12.8	58	14.8	13	18.3	
Histology	NS	383	69.1	284	72.6	99	60.7	0.001
	Lobular	103	18.6	56	14.3	47	28.8	
	Mixt	20	3.6	13	3.3	7	4.3	
	Others	35	6.3	29	7.4	6	3.7	
	Micro-invasive	13	2.3	9	2.3	4	2.5	
Grade	1	109	19.7	87	22.3	22	13.5	0.096
	2	304	54.9	206	52.7	98	60.1	
	3	111	20.0	80	20.5	31	19.0	
	Missing	30	5.4	18	4.5	12	7.4	
pT size	pT1	248	45.6	181	46.9	67	42.4	0.557
	pT2	214	39.3	150	38.9	64	40.5	
	pT3	82	15.1	55	14.2	27	17.1	
LVI	No	327	59.0	222	56.8	105	64.4	0.180
	Yes	179	32.3	131	33.5	48	29.4	
	Missing	48	8.7	38	9.7	10	6.1	
ER status	ER+	507	91.5	360	92.1	147	90.2	0.689
	ER-	37	6.7	25	6.4	12	7.4	
	Missing	10	1.8	6	1.5	4	2.5	
Subtypes	ER+ Her2-	390	83.5	282	84.9	108	80.0	0.253
	ER- Her2-	13	2.8	10	3.0	3	2.2	
	ER+ Her2+	45	9.6	30	9.0	15	11.1	
	ER- Her2+	19	4.1	10	3.0	9	6.7	
	Missing	87		59		28		
Axillary surgery	SLNB	183	33.0	111	28.4	72	44.2	<0.0001
	cALND	371	67.0	280	71.6	91	55.8	
pN status	pN1	52	9.4	44	11.3	8	4.9	0.018
	pN1mi or pN0(i+)	502	90.6	347	88.7	155	95.1	
Chemotherapy	No	234	42.2	150	38.4	84	51.5	0.017
	Yes	303	54.7	228	58.3	75	46.0	
	NAC	17	3.1	13	3.3	4	2.5	
Radiotherapy	No	195	35.2	120	30.7	75	46.0	0.001
	Yes	359	64.8	271	69.3	88	54.0	
Endocrine therapy	No	26	5.1	14	3.9	12	8.2	0.049
for ER+	Yes	480	94.9	345	96.1	135	91.8	
Trastuzumab	No	502	90.6	356	91.0	146	89.6	0.587
	Yes	52	9.4	35	9.0	17	10.4	
Death	No	525	94.8	372	95.1	153	93.9	0.335
	Yes	29	5.2	19	4.9	10	6.1	
Recurrence	No	509	91.9	362	92.6	147	90.2	0.218
	Yes	45	8.1	29	7.4	16	9.8	
Axillary recurrence	No	549	99.1	387	99.0	162	99.4	0.700
	Yes	5	0.9	4	1.0	1	0.6	
Metastases	No	529	95.5	374	95.7	155	95.1	0.772
	Yes	25	4.5	17	4.3	8	4.9	

Legend: NS: nonspecific, LVI: lympho-vascular invasion, ER: endocrine receptor, SLNB: sentinel lymph node biopsy, cALND: completion axillary lymph node dissection, and NAC: neo-adjuvant chemotherapy.

**Table 2:** Characteristics of 554 patients according to cALND or SLNB alone.

		All Patients			Chi 2
	cALND	No	Yes	%	*p*
All patients		183	371	67.0	
pN sn	pN0(i+)	72	91	55.8	<0.0001
	pN1mi	111	280	71.6	
Age	≤40	28	53	65.4	<0.0001
	41–50	38	101	72.7	
	51–74.9	65	198	75.3	
	≥75	52	19	26.8	
Histology	NS	128	255	66.6	0.475
	Lobular	38	65	63.1	
	Mixt	6	14	70.0	
	Others	7	28	80.0	
	Micro-invasive	4	9	69.2	
Grade	1	22	87	79.8	0.029
	2	107	197	64.8	
	3	42	69	62.2	
	Missing	12	18	61.5	
pT size	pT1	77	171	69.0	0.041
	pT2	83	131	61.2	
	pT3	20	62	75.6	
LVI	No	128	199	60.9	<0.0001
	Yes	48	131	73.2	
	Missing	7	41	85.4	
ER status	ER+	172	335	66.1	0.301
	ER−	8	29	78.4	
	Missing	3	7	70.0	
Subtypes	ER+ Her2−	147	243	62.3	0.158
	ER− Her2−	2	11	84.6	
	ER+ Her2+	14	31	68.9	
	ER− Her2+	4	15	78.9	
Chemotherapy	No	120	114	48.7	<0.0001
	Yes	62	241	79.5	
	NAC	1	16	94.1	
Radiotherapy	No	99	96	49.2	<0.0001
	Yes	84	275	76.6	
Endocrine therapy	No	10	16	61.5	0.382
for ER+	Yes	162	318	66.2	
Trastuzumab	No	172	330	65.7	0.036
	Yes	11	41	78.8	
Death	No	171	354	67.4	0.326
	Yes	12	17	58.6	
Recurrence	No	167	342	67.2	0.707
	Yes	16	29	64.4	
Axillary recurrence	No	182	367	98.9	0.534
	Yes	1	4	1.1	
Metastases	No	173	356	96.0	0.448
	Yes	10	15	4.0	

Legend: NS: nonspecific, LVI: lympho-vascular invasion, ER: endocrine receptor, cALND: completion axillary lymph node dissection, NAC: neo-adjuvant chemotherapy, and sn: sentinel node. For patients with cALND, NSN involvement rates with macrometastases were 13.2% (49/371): 7.7% (7/91) and 15.0% (42/280) for pN0(i+) and pN1mi, respectively (*p* < 0.0001). Significant factors associated with cALND and with radiotherapy.

**Table 3:** Significant factors associated with cALND in regression analysis.

	cALND	Nb	*p*	OR	CI 95%	
					Inferior	Superior
**Grade**	Grade 1	109	0.002	**1**		
	**Grade 2**	304	**0.004**	**0.419**	0.230	0.764
	**Grade 3**	111	**<0.0001**	**0.212**	0.099	0.453
**SN status**	**pN1mi vs. pN0(i+)**		**0.013**	**1.773**	1.129	2.785
Age	≤40	80	<0.0001	**1**		
	40.1–50	136	0.085	1.838	0.919	3.674
	**50.1–74.9**	257	**0.007**	**2.392**	1.273	4.495
	≥75	71	0.091	0.488	0.212	1.123
pT size	pT1	248	0.228	**1**		
	pT2	214	0.090	0.670	0.421	1.064
	pT3	82	0.392	0.742	0.374	1.469
**LVI**	No LVI	326	0.018	**1**		
	**LVI**	179	**0.034**	**1.697**	1.040	2.769
	Unknown	39	0.033	3.208	1.100	9.354
**Radiotherapy**	**No vs. yes**		0.006	0.511	0.316	0.828
**Chemotherapy**	No AC	225	<0.0001	**1**		
	**AC**	302	<0.0001	3.548	2.052	6.137
	**NAC**	17	0.021	12.103	1.462	100.160

Legend: LVI: lympho-vascular invasion, cALND: completion axillary lymph node dissection, AC: adjuvant chemotherapy, and NAC: neo-adjuvant chemotherapy. Significant factors are report in bold character.

**Table 4:** Significant factors associated with radiotherapy.

	Radiotherapy	*p*	OR	CI 95%	
				Inferior	Superior
**cALND**	**No vs. Yes**	**0.006**	**0.503**	0.308	0.823
Grade	Grade 1	0.178	1		
	Grade 2	0.786	1.080	0.619	1.884
	Grade 3	0.986	1.007	0.475	2.131
**Chemotherapy**	No	<0.0001	1		
	**AC**	**<0.0001**	**5.757**	3.440	9.636
	**NAC**	**0.015**	**13.485**	1.655	109.843
Age	≤40	0.995	1		
	40.1–50	0.997	0.998	0.463	2.151
	50.1–74.9	0.859	1.068	0.520	2.192
	≥75	0.940	1.035	0.423	2.536
**pT size**	pT1	<0.0001	1		
	pT2 < 30 mm	0.903	1.034	0.602	1.776
	pT2 ≥ 30 mm	0.312	1.365	0.747	2.494
	**pT 3**	**<0.0001**	**7.858**	3.107	19.875
**LVI**	No	0.064	1		
	**LVI**	**0.021**	**1.796**	1.091	2.954
	Unknown	0.396	1.470	0.604	3.577
**SN status**	**pN1mi vs. pN0(i+)**	**0.037**	**1.673**	1.030	2.715

Legend: LVI: lympho-vascular invasion, cALND: completion axillary lymph node dissection, SN: sentinel node, AC: adjuvant chemotherapy, and NAC: neo-adjuvant chemotherapy. Significant factors are report in bold character.

**Table 5:** Results of OS, DFS, RFS and MFS in univariate analysis for all patients, and for SLNB alone and SLNB with cALND.

Kaplan–Meier		2 years	5 years	7 years	10 years	Log Rank
**OS**	%	98.8	97.5	95.8	92.5	
	SD	0.5	0.7	1.1	1.8	
	Nb at risk	478	324	184	98	
OS SLNB	%	97.5	96.5	93.6	86.7	0.002
	SD	1.2	1.6	3.2	5.6	
	Nb at risk	134	69	32	16	
OS cALND	%	99.4	98.1	96.7	94.0	
	SD	0.4	0.8	1.1	1.9	
	Nb at risk	343	254	151	82	
**DFS**	%	98.1	93.7	90.4	87.3	
	SD	0.6	1.2	1.6	2.1	
	Nb at risk	473	310	171	88	
DFS SLNB	%	95.7	91.4	83.8	72.2	0.001
	SD	1.6	2.4	4.8	7.5	
	Nb at risk	131	65	28	11	
DFS cALND	%	99.2	94.9	92.5	90.9	
	SD	0.5	1.3	1.6	1.9	
	Nb at risk	341	244	142	77	
**RFS**	%	98.7	94.3	91.7	89.6	
	SD	0.5	1.1	1.5	1.9	
	Nb at risk	473	310	172	89	
RFS SLNB	%	97.0	92.7	85.0	80.2	0.003
	SD	1.3	2.3	4.8	6.5	
	Nb at risk	131	65	28	11	
RFS cALND	%	99.4	95.1	93.6	92.0	
	SD	0.4	1.2	1.5	1.9	
	Nb at risk	341	244	143	78	
**MFS**	%	98.5	95.5	93.8	89.5	
	SD	0.5	1.0	1.3	2.0	
	Nb at risk	475	315	176	90	
MFS SLNB	%	96.4	92.9	88.0	75.9	0.001
	SD	1.4	2.2	4.1	7.4	
	Nb at risk	132	66	28	11	
MFS cALND	%	99.4	96.7	95.7	92.7	
	SD	0.4	1.0	1.2	1.9	
	Nb at risk	342	248	147	79	
**BCSS**	%	99.4	98.1	96.8	94.5	
	SD	0.3	0.7	1.0	1.6	
	Nb at risk	478	324	184	98	
BCSS SLNB	%	98.9	97.8	94.9	94.9	0.021
	SD	0.8	1.3	3.1	3.1	
	Nb at risk	134	69	32	16	
BCSS cALND	%	99.7	98.4	97.3	94.7	
	SD	0.3	0.7	1.0	1.8	
	Nb at risk	343	254	151	82	

Legend: DFS: disease-free survival, OS: overall survival, RFS: recurrence-free survival, MFS: metastases-free survival, BCSS: breast-cancer specific survival, SLNB: sentinel lymph node biopsy, and cALND: completion axillary lymph node dissection.

**Table 6:** Survival results for all patients in multivariate analysis.

All Patients	DFS			OS			RFS			MFS			BCSS		
	HR	CI 95%	*p*	HR	CI 95%	*p*	HR	CI 95%	*p*	HR	CI 95%	*p*	HR	CI 95%	*p*
SLNB and cALND	**1**			**1**			**1**			**1**			1		
SLNB alone	**2.538**	**1.276–5.049**	**0.008**	**2.583**	**1.031–6.473**	**0.043**	**2.565**	**1.204–5.463**	**0.015**	**2.756**	**1.228–6.183**	**0.014**	2.760	0.953–7.993	0.061
pN1mi vs. pN0(i+)	0.662	0.361–1.216	0.184	1.006	0.432–2.344	0.989	0.762	0.385–1.506	0.434	0.742	0.362–1.521	0.415	1.275	0.453–3.583	0.645
Grade 1	1			1			1			1			1		
Grade 2	0.803	0.383–1.687	0.563	1.411	0.487–4.086	0.525	0.857	0.364–2.018	0.725	1.251	0.482–3.246	0.646	1.916	0.509–7.212	0.336
Grade 3	1.133	0.440–2.916	0.796	2.816	0.765–10.361	0.119	1.036	0.361–2.968	0.948	1.879	0.602–5.865	0.278	2.688	0.558–12.944	0.218
≤40 years	1			1			1			1			1		
41–50	1.080	0.462–2.524	0.859	1.035	0.299–3.580	0.956	0.844	0.330–2.159	0.723	1.710	0.508–5.757	0.386	0.553	0.123–2.492	0.441
51–74.9	0.998	0.448–2.225	0.996	1.616	0.518–5.036	0.408	0.951	0.405–2.231	0.908	1.798	0.551–5.860	0.331	1.648	0.507–5.361	0.406
≥75	0.549	0.138–2.187	0.395	0.774	0.130–4.593	0.778	0.422	0.083–2.142	0.298	1.140	0.229–5.687	0.873	0.526	0.053–5.256	0.584
No RTH vs. RTH	1.761	0.852–3.640	0.127	1.543	0.567–4.201	0.396	**2.342**	**1.047–5.239**	**0.038**	1.421	0.608–3.321	0.417	2.176	0.660–7.178	0.202
No AC	1			1			1			1			1		
AC	0.358	0.080–1.598	0.178	0.508	0.173–1.492	0.218	1.597	0.653–3.908	0.305	1.121	0.435–2.890	0.814	0.922	0.263–3.235	0.899
NAC	0.353	0.096–1.296	0.117	0.772	0.077–7.753	0.826	**5.389**	**1.109–26.186**	**0.037**	1.927	0.210–17.670	0.562	1.395	0.122–15.968	0.789
LVI vs. no LVI	0.894	0.449–1.777	0.748	0.570	0.214–1.518	0.261	0.885	0.419–1.871	0.749	0.610	0.271–1.373	0.232	0.639	0.212–1.921	0.639

Legend: DFS: disease-free survival, OS: overall survival, RFS: recurrence-free survival, MFS: metastases-free survival, BCSS: breast-cancer specific survival, LVI: lympho-vascular invasion, SLNB: sentinel lymph node biopsy, cALND: completion axillary lymph node dissection, AC: adjuvant chemotherapy, NAC: neo-adjuvant chemotherapy, and RTH: radiotherapy. Significant factors are report in bold character.

**Table 7:** Survival results for Her2-positive or triple-negative breast cancer patients in multivariate analysis.

Her2-Positive	DFS			OS			RFS		
& TNBC	HR	CI 95%	*p*	HR	CI 95%	*p*	HR	CI 95%	*p*
SLNB and cALND	**1**						**1**		
SLNB alone	**38.451**	**1.437–1028.75**	**0.030**	1.971	0.118–32.840	0.636	**38.451**	**1.437–1028.75**	**0.030**
pN1mi vs. pN0(i+)	4.398	0.359–53.880	0.247	1.056	0.125–8.960	0.960	4.398	0.359–53.880	0.247
Grade 1–2	1						1		
Grade 3	0.048	0.003–0.858	0.039	0.139	0.013–1.500	0.104	0.048	0.003–0.858	0.039
≤50 years	1						1		
>50 years	2.314	0.299–17.922	0.422	8.001	0.561–114.043	0.125	2.314	0.299–17.922	0.422
No RTH vs. RTH	**7.824**	**1.246–49.118**	**0.028**	3.913	0.497–30.785	0.195	**7.824**	**1.246–49.118**	**0.028**
No chemotherapy	1						1		
AC			0.973	2.565	0.148–44.411	0.517			0.973
NAC			0.996						0.996
LVI vs. no LVI	2.308	0.316–16.849	0.410	0.711	0.081–6.233	0.758	2.308	0.316–16.849	0.410

Legend: DFS: disease-free survival, OS: overall survival, RFS: recurrence-free survival, LVI: lympho-vascular invasion, SLNB: sentinel lymph node biopsy, cALND: completion axillary lymph node dissection, AC: adjuvant chemotherapy, NAC: neo-adjuvant chemotherapy, and RTH: radiotherapy. Significant factors are report in bold character.

**Table 8:** Survival results for ER-positive Her2-negative breast cancer patients in multivariate analysis.

ER-Positive	DFS			OS			RFS			MFS			BCSS		
Her2-Negative	HR	CI 95%	*p*	HR	CI 95%	*p*	HR	CI 95%	*p*	HR	CI 95%	*p*	HR	CI 95%	*p*
SLNB and cALND	**1**			1			1			1			1		
SLNB alone	**2.358**	**1.027–5.414**	**0.043**	**3.317**	**1.054–10.439**	**0.040**	**2.538**	**1.005–6.414**	**0.049**	2.571	0.963–6.861	0.059	3.517	0.927–13.348	0.065
pN1mi vs. pN0(i+)	0.608	0.290–1.275	0.188	1.111	0.372–3.319	0.851	0.640	0.279–1.472	0.294	0.581	0.239–1.414	0.231	1.291	0.342–4.868	0.707
Grade 1	1			1											
Grade 2	0.596	0.246–1.445	0.252	0.566	0.156–2.051	0.386	0.618	0.227–1.686	0.347	0.584	0.191–1.781	0.344	0.945	0.198–4.507	0.944
Grade 3	1.118	0.375–3.337	0.842	2.139	0.486–9.419	0.315	1.036	0.299–3.581	0.956	2.004	0.573–7.010	0.277	2.247	0.349–14.450	0.394
≤40 years	1			1											
41–50	0.835	0.286–2.439	0.742	0.786	0.161–3.835	0.766	0.635	0.189–2.139	0.464	1.049	0.230–4.773	0.951	0.546	0.081–3.694	0.535
51–74.9	0.821	0.305–2.207	0.696	1.238	0.282–5.435	0.777	0.766	0.264–2.224	0.624	1.412	0.340–5.868	0.635	1.306	0.258–6.610	0.747
≥75	0.696	0.156–3.110	0.635	0.842	0.111–6.384	0.868	0.539	0.093–3.121	0.490	1.386	0.220–8.740	0.728	0.655	0.051–8.488	0.746
No RTH vs. RTH	1.241	0.506–3.041	0.637	1.012	0.298–3.438	0.984	1.138	0.405–3.203	0.806	0.658	0.228–1.898	0.439	0.748	0.148–3.767	0.724
No AC	1			1											
AC	0.856	0.334–2.198	0.747	**0.271**	**0.075–0.978**	**0.046**	1.030	0.351–3.020	0.957	0.622	0.205–1.892	0.403	0.340	0.076–1.531	0.160
NAC	4.542	0.729–28.294	0.105	0.982	0.071–13.622	0.989	**8.232**	**1.223–55.409**	**0.030**	4.952	0.455–53.933	0.189	1.355	0.090–20.395	0.826
LVI vs. no LVI	0.780	0.336–1.814	0.564	0.738	0.104–5.262	0.762	0.665	0.258–1.716	0.399	0.527	0.182–1.522	0.237	0.882	0.199–3.921	0.869

Legend: DFS: disease-free survival, OS: overall survival, RFS: recurrence-free survival, MFS: metastases-free survival, BCSS: breast-cancer specific survival, LVI: lympho-vascular invasion, SLNB: sentinel lymph node biopsy, cALND: completion axillary lymph node dissection, AC: adjuvant chemotherapy, NAC: neo-adjuvant chemotherapy, and RTH: radiotherapy. Significant factors are report in bold character.

**Table 9:** Survival results for ER-positive Her2-negative breast cancer patients in multivariate analysis, according to age ≤ 50 years or >50 years.

	DFS			OS			RFS			MFS			BCSS		
	HR	CI 95%	*p*	HR	CI 95%	*p*	HR	CI 95%	*p*	HR	CI 95%	*p*	HR	CI 95%	*p*
**ER-positive Her2-negative** **≤50 years**															
cALND	**1**			1			1			1			1		
SLNB alone	**3.185**	**0.890–11.402**	**0.075**	**103.47**	**4.583–2335.8**	**0.004**	2.644	0.587–11.913	0.206	4.509	0.829–24.536	0.081	**50.874**	**1.330–1945.4**	**0.035**
pN1mi vs. pN0(i+)	0.581	0.170–1.984	0.386	8.094	0.566–115.76	0.123	0.701	0.184–2.672	0.603	0.807	0.142–4.589	0.809	3.402	0.175–66.096	0.419
Grade 1	1			1											
Grade 2	0.395	0.122–1.283	0.122	**0.055**	**0.005–0.614**	**0.018**	0.375	0.094–1.502	0.166	0.315	0.059–1.671	0.175	0.084	0.004–1.651	0.103
Grade 3	0.163	0.017–1.601	0.120	0.284	0.014–5.796	0.413	0.243	0.023–2.574	0.240	0.218	0.020–2.374	0.211	1.306	0.051–33.331	0.872
No RTH vs. RTH	**3.948**	**1.016–15.335**	**0.047**	**10.904**	**1.410–84.315**	**0.022**	4.006	0.898–17.863	0.069	1.451	0.247–8.508	0.680	9.660	0.987–94.587	0.051
No AC	1			1											
AC	1.890	0.442–8.075	0.390	1.538	0.149–15.908	0.718	1.059	0.201–5.583	0.946	1.991	0.268–14.794	0.501	0.920	0.040–21.254	0.959
NAC	5.405	0.424–68.96	0.194				4.677	0.297–73.775	0.273			0.994			0.994
LVI vs. no LVI	1.665	0.484–5.726	0.418	**10.804**	**0.833–140.21**	**0.022**	2.136	0.498–9.171	0.307	0.742	0.131–4.200	0.736	33.475	0.874–1281.8	0.059
**ER-positive Her2-negative** **>50 years**															
cALND	1														
SLNB alone	1.321	0.371–4.699	0.667	1.164	0.231–5.880	0.854	2.278	0.514–10.087	0.278	1.584	0.389–6.448	0.521	2.350	0.157–35.249	0.536
pN1mi vs. pN0(i+)	1.087	0.336–3.524	0.889	2.784	0.456–16.991	0.267	1.505	0.374–6.061	0.565	0.741	0.220–2.496	0.629	1.328	0.114–15.504	0.821
Grade 1	1														
Grade 2	1.100	0.268–4.509	0.894	2.267	0.314–16.359	0.417	1.733	0.380–7.916	0.478	1.154	0.243–5.484	0.857	2.616	0.195–35.089	0.468
Grade 3	**11.499**	**2.293–57.656**	**0.003**	**20.624**	**1.893–224.67**	**0.013**	**13.270**	**1.972–89.286**	**0.008**	**8.163**	**1.464–45.516**	**0.017**	2.936	0.085–101.85	0.552
No RTH vs. RTH	0.410	0.097–1.737	0.226	0.415	0.065–2.656	0.353	0.145	0.015–1.423	0.098	0.462	0.105–2.029	0.306	0.276	0.001–62.698	0.642
No AC7	1														
AC	0.387	0.113–1.323	0.130	**0.072**	**0.011–0.488**	**0.007**	0.700	0.139–3.520	0.665	0.320	0.075–1.364	0.13	0.370	0.020–6.939	0.506
NAC	**38.199**	**4.750–307.21**	**0.001**	8.582	0.524–140.543	0.132	**113.84**	**7.372–1758.061**	**0.001**	**14.806**	**0.944–232.19**	**0.055**	**655.305**	**5.870–73** **,** **161**	**0.007**
LVI vs. no LVI	**0.132**	**0.026–0.664**	**0.014**	0.127	0.011–1.451	0.097	**0.043**	**0.004–0.448**	**0.008**	0.228	0.043–1.212	0.083	0.330	0.029–3.753	0.371

Legend: DFS: disease-free survival, OS: overall survival, RFS: recurrence-free survival, MFS: metastases-free survival, BCSS: breast-cancer specific survival, LVI: lympho-vascular invasion, SLNB: sentinel lymph node biopsy, cALND: completion axillary lymph node dissection, AC: adjuvant chemotherapy, NAC: neo-adjuvant chemotherapy, and RTH: radiotherapy. Significant factors are report in bold character.

## Data Availability

Data supporting reported results can be found in Paoli Calmettes Institute breast cancer database.
